# Comparison of Floseal^®^ and Tranexamic Acid for Bleeding Control after Total Knee Arthroplasty: a Prospective Randomized Study

**DOI:** 10.6061/clinics/2019/e1186

**Published:** 2019-11-11

**Authors:** Camilo Partezani Helito, Marcelo Batista Bonadio, Marcel Faraco Sobrado, Pedro Nogueira Giglio, José Ricardo Pécora, Gilberto Luis Camanho, Marco Kawamura Demange

**Affiliations:** I Departamento de Ortopedia e Traumatologia, Hospital das Clinicas HCFMUSP, Faculdade de Medicina, Universidade de Sao Paulo, Sao Paulo, SP, BR; II Hospital Sirio Libanes, Sao Paulo, SP, BR

**Keywords:** Total Knee Arthroplasty, Bleeding Control, Tranexamic Acid, Hemostatic, Transfusion Rate

## Abstract

**OBJECTIVE:**

Tranexamic acid (TXA) and the hemostatic agent Floseal^®^ have already been used to minimize bleeding during total knee arthroplasty (TKA).

**METHODS:**

We conducted a prospective, randomized study of 90 patients with indications for TKA. Following inclusion, the participants were randomly allocated in blocks to the following 3 groups: control, Floseal^®^ and TXA. Bleeding parameters, including decreases in hemoglobin (Hb), drain output, number of blood transfusions and complications, were assessed. ClinicalTrials.gov: NCT02152917.

**RESULTS:**

The mean decrease in Hb was highest in the control group (4.81±1.09 g/dL), followed by the Floseal^®^ (3.5±1.03 g/dL) and TXA (3.03±1.2 g/dL) groups. The Floseal^®^ and TXA groups did not differ, and both performed better than the control group. The mean total drain output was 901.3±695.7 mL in the control group, 546.5±543.5 mL in the TXA group and 331.2±278.7 mL in the Floseal^®^ group. Both TXA and Floseal^®^ had significantly less output than the control group, and Floseal^®^ had significantly less output than TXA. The number of blood transfusions was very small in all 3 groups.

**CONCLUSION:**

The use of TXA or Floseal^®^ was associated with less blood loss than that of the control group among patients undergoing primary TKA, as measured both directly (intraoperative bleeding + drainage) and on the basis of a decrease in Hb, without differences in the rate of complications. TXA and Floseal^®^ showed similar decreases in Hb and total measured blood loss, but the drain output was smaller in the Floseal^®^ group.

## INTRODUCTION

The number of knee arthroplasty (TKA) procedures performed per year is expected to gradually increase as a function of population aging and increases in life expectancy. According to American estimates, the annual number of procedures will increase to 3 million by 2030 (1,2). Although TKA is well established as a treatment for knee arthritis, several issues relating to the perioperative clinical control of patients still remain, particularly regarding complications associated with clinical comorbidities and surgery-induced bleeding.

Tranexamic acid (TXA) has been suggested as a therapeutic option to minimize blood loss. Several studies have reported excellent results with the intravenous administration of TXA during the perioperative period in terms of bleeding reduction, decreases in hemoglobin (Hb) levels and a decreased need for blood transfusions (1-6). However, the association of TXA with thrombotic events is a cause of much concern, and consequently, most studies have excluded patients with comorbidities that are likely to increase the risk of thrombosis. Whiting et al. (7) assessed the use of TKA in high-risk patients and did not observe an increase in thromboembolic events. However, theirs was a retrospective study, and thus, it is not yet possible to assert whether TXA is completely safe for this population of patients. Most surgeons avoid using TXA when there is a higher perceived risk of thrombotic events, such as in patients with a history of venous thromboembolism or ischemic cardiovascular events.

Another approach to minimize bleeding is based on the use of topical hemostatic agents, such as Floseal^®^ (Baxter). This compound is composed of human thrombin and bovine-derived gelatin and is designed for topical use (8). Some studies have reported significant benefits of using hemostatic agents to control bleeding in several medical areas (9-12), including orthopedic surgery (13-18), but its action has not yet been well established in TKA.

To the best of our knowledge, no studies have compared TXA and Floseal^®^. Therefore, it is not yet known whether their effects on bleeding control are comparable and thus whether Floseal^®^ may be used in place of TXA in cases of contraindication. Therefore, we conducted a randomized, prospective study comprising 3 groups (TXA, Floseal^®^ and control) to first compare the efficacy of TXA and a hemostatic agent for controlling perioperative bleeding in TKA, using direct measurement (intraoperative bleeding + drain output) and a decrease in the Hb concentration, and secondarily to assess the respective rates of complications. Our hypotheses were that both treatments would be more efficient than the control treatment and that TXA would be superior to the topical hemostatic agent.

## MATERIALS AND METHODS

The present prospective, randomized study was conducted with an initial population of 90 patients with indications for TKA. The study was approved by the institutional research ethics committee, and all of the participants signed informed consent forms. The protocol was registered at clinicaltrials.gov under NCT02152917. The inclusion criteria are described in Table 1, and these criteria were selected to avoid including patients with high odds of bleeding or postoperative (PO) complications.

Following inclusion, the participants were randomly allocated in blocks to the following 3 groups: control, Floseal^®^ and TXA.

All TKA procedures were performed in the standard manner using pneumatic tourniquets and the medial parapatellar and transquadricipital approach without patellar eversion. An intramedullary guide was used for the femoral cut, and an extramedullary guide was used for the tibial cut. The posterior cruciate ligament was resected, and the patellar cut was performed in all cases. Cement fixation was performed on all patients, none of whom received any type of periarticular infiltration. In all patients, the pneumatic tourniquet was released after cementation. A compressive dressing was applied to the open wound for 5 minutes after the release of the tourniquet, and electrocautery was used as needed before closure.

The anesthetist was the only member of the surgical staff who had knowledge of the group the participants belonged to before and during surgery and was responsible for the administration of TXA. In the Floseal^®^ group, the surgeon was informed and handled the product for application at the time of pneumatic tourniquet release (when the surgeon was informed of the group to which the patient was allocated). The participants in the control group received no other treatments than those usually administered during surgery.

**Figure 1:**
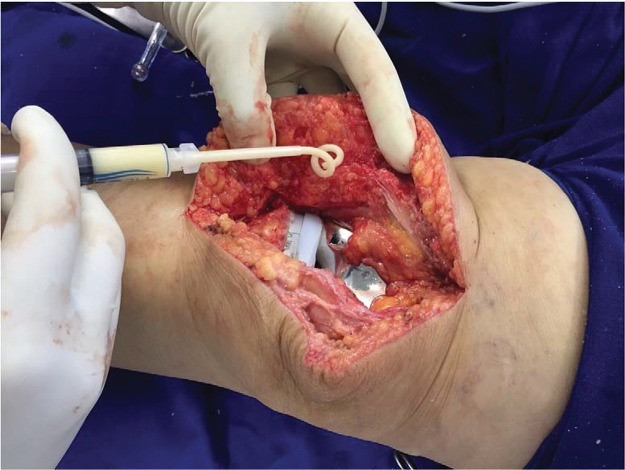
Application of Floseal^®^ to the surgical wound.

Floseal^®^ was applied to the potential bleeding sites, particularly the posterior, superior, medial and lateral recesses, before the pneumatic tourniquet was released and the surgical approach was then tamponed with a compressive dressing (Figure 1). After the tamponed period (five minutes), the product was again applied to the bleeding sites with a bandage at those points for two minutes. A total of 10 mL of Floseal^®^ was used, and it was distributed into 2 syringes (5 mL in each).

TXA was administered at a dose of 10 mg/kg at least 20 minutes before inflating the pneumatic tourniquet, and another 10 mg/kg was administered before tourniquet release. In the present study, we chose the dose and simplest administration route for TXA from those reported in the literature as being able to reduce bleeding and the need for transfusion (1).

The follow-up time had a minimum of 12 months, and all the parameters assessed are described in Table 2.

All participants received standard mechanical and pharmacological prophylaxis against thromboembolism with 40 mg of enoxaparin once daily for 30 days, as it is the standard protocol at our institution. In addition, they received oxygen via a catheter for 24 hours after surgery because of its beneficial effects on wound healing according to recent literature (19). The drain was removed on PO day 2, within 46 to 48 hours after the procedure, and exercises targeting range of motion and walking were started the day after surgery.

Statistical analysis was performed using Pearson’s chi-square test or Fisher’s test for categorical variables. Continuous variables were described by the mean and standard deviation, and Shapiro-Wilk tests and histograms were used to check the normality of the data. We used a one-way analysis of variance (ANOVA) test for normally distributed variables and the Kruskal-Wallis test for non-normally distributed variables, as well as a post hoc test with Bonferroni correction. An a priori sample size estimation of 90 patients per group was chosen on the basis of a power of 80% to show a clinically significant difference of 500 ml of total bleeding between the intervention groups and the control group (20). We used the statistical software SPSS 22 (IBM Corp., NY, USA) and G*Power 3.1.9.3 (Erdfelder, Faul, Buchner, Universität Düsseldorf, Düsseldorf, Germany 2009).

## RESULTS

A total of 90 patients were included in the study, with 30 allocated to each group, and none of the participants were lost to follow-up during the study period. The mean values of the investigated variables are described in Table 3.

The mean decrease in Hb was largest in the control group (4.81±1.09 g/dL), followed by the Floseal^®^ (3.5±1.03 g/dL) and TXA (3.03±1.2 g/dL) groups (*p*<0.001). The hemostatic and TXA groups did not differ in this parameter (*p*=0.286), and both treatments showed a lower performance than the control treatment (*p*<0.05).

The mean drain output was 901.3±695.7 mL in the control group, 546.5±543.5 mL in the TXA group and 331.2±278.7 mL in the Floseal^®^ group (*p*<0.001). The drain output was significantly higher in the control group than in the TXA (*p*<0.05) and Floseal^®^ (*p*<0.05) groups (*p*<0.001) and was higher in the TXA group than in the Floseal^®^ group (*p*=0.016).

The amount of directly aspirated blood was different between the three groups (*p*=0.001). Both the TXA and Floseal^®^ groups had less aspirated blood than the control group (*p*=0.001 and 0.05, respectively). The pad weight did not differ between the TXA and Floseal^®^ groups (*p*=0.46) and was smaller in these groups than in the control group (*p*<0.05). The amount of infused serum did not differ among the groups (*p*=0.53).

The total bleeding volume (aspirated blood + pad weight difference + drain output on PO day 2) was different between the three groups (*p*<0.001). It was higher in the control group than in the TXA (1328 ml *vs*. 799 ml, *p*=0.001) and Floseal^®^ (1328 ml *vs*. 626 ml, *p*<0.05) groups, and there was no statistically significant difference between TXA and Floseal^®^ (799 ml *vs*. 626 ml, *p*<0.001).

The number of blood transfusions was very small, with only 1 patient from the Floseal^®^ and TXA groups and 2 patients from the control group requiring transfusion. Complications included 1 case of superficial infection and 1 case of deep vein thrombosis (DVT) in the control group, 1 case of deep infection treated by debridement and polyethylene exchange in the TXA group and 1 case of DVT in the Floseal^®^ group. All complications were detected within the first 3 months after surgery.

The results concerning range of motion (ROM) of the knees are described in Table 4. The ROM was higher in the Floseal^®^ group than in the control group on all 3 of the PO days (*p*<0.05). The ROM was also better in the Floseal^®^ group than in the TXA group on PO days 2 and 3 (*p*<0.05), but it did not differ on PO day 1 (*p*=0.13). Comparing the TXA and control groups, the ROM was similar on PO day 1 (*p*=0.11) and better in the TXA group on the 2 following days (*p*<0.05). The groups did not differ in terms of gains in ROM during the first 3 days after surgery.

## DISCUSSION

The results of the present study corroborate previous reports in the literature regarding the reduction of bleeding by TXA in TKA and confirm the efficacy of the hemostatic agent Floseal^®^ (6,17,18,21-25). In the present study, the use of Floseal^®^ was associated with a smaller decrease in Hb levels than that in the control (3.50 g/dL *vs*. 4.81 g/dL), this decrease was not significantly different from that with TXA (3.50 g/dL *vs*. 3.03). There are already published reports of reductions in bleeding with the use of hemostatic agents (26-29). However, there is considerable disagreement in the literature, as there are also several studies with satisfactory methodology that have not detected this difference (16,30,31).

Similar to those in another study (18), the rate of complications in our investigation, including infections and venous thromboembolism, was small and did not differ among the groups. Patients with severe comorbidities and risk factors for thromboembolic complications were excluded from the present study, and therefore, we did not expect adverse events to have a high occurrence. Although Whiting et al. (7) did not find an association between the use of TXA and an increased frequency of thromboembolic events among high-risk patients, this medication is not commonly used in this group of patients in our country, and there is still a tendency worldwide to avoid using TXA in the presence of a higher risk of thromboembolic events.

One interesting finding of our study was that the initial ROM of the knee was higher among the patients who received Floseal^®^. This parameter is not frequently assessed in studies investigating the use of hemostatic and fibrinolytic agents, but we believe it is of paramount importance because the effects of these blood-loss management approaches on the patients’ rehabilitation should be taken into consideration. In addition to causing eventual clinical complications, joint bleeding after TKA can impair the ROM of the knee due to the presence of joint effusion, which causes pain and impairs mobility. Although in the present study all of the groups attained an average ROM of 90 degrees or more by PO day 3, the hemostatic group exhibited a higher ROM on all 3 days, and this was the only group to attain more than 90 degrees on PO day 2 and more than 100 degrees on PO day 3; these differences were statistically significant compared with the control and TXA groups.

To the best of our knowledge, our study is the first to include a group treated with TXA in addition to the control group and to compare TXA to treatment with a topical hemostatic agent. The results corresponding to both intervention groups (Floseal^®^ and TXA) were superior to those from the control group and were not different in terms of reduced bleeding and a reduction of the decrease in Hb, and both interventions were accompanied by similarly low rates of complications. Therefore, we conclude that Floseal^®^ is a viable resource for reducing bleeding in primary TKA, with effects that are comparable to the more well-known TXA treatment. The only factor that remains to be established is the cost-benefit ratio, which was not assessed in the present study.

Topical TXA has recently been further studied. Recent studies have reported results that were similar to the use of intravenous TXA, with less potential for complications (32,33). Future studies comparing topical TXA and hemostatic agents can now be conducted, as both treatments were found to be equivalent to intravenous TXA. We believe that all of these agents should be part of the therapeutic arsenal to minimize perioperative bleeding so that patients exhibiting contraindications to one of the agents might benefit from the others. In cases with no contraindications, the choice may be combined use or use of the agent with the best cost-benefit ratio for the healthcare service.

In addition, we consider investigating the possible benefits of the combined use of TXA and hemostatic agents to be relevant because they have different mechanisms of action and their effects might thus be potentiated. The combined approach might be beneficial considering the recent trend toward a reduction in the lengths of hospital stays. Rehabilitation of patients subjected to TKA can be initiated earlier, and the use of postoperative drains can occasionally be avoided.

The present study has certain limitations. Although the surgeon was blinded to the randomization and was unaware if the patient received the initial application of TXA during surgery, blinding was not possible in the case of the hemostatic agent, as it is applied by the surgeon during the surgical procedure. This might have resulted in some bias because the surgeon was aware of the cases in which the hemostatic agent was used at the end of hemostasis. Additionally, we could not reject the null hypothesis that the total bleeding volume was the same between the TXA and Floseal^®^. As our post hoc analysis showed that a minimal sample size of 124 subjects per group would be required to achieve a power of 0.8, there is a possibility of a type II error regarding this finding. Nevertheless, in our judgment, the observed difference of 174 ml was clinically too small to justify the increase in sample size. Based on the results of the present study, future trials should be conducted with topical TXA to compare both topical treatments only.

## CONCLUSION

The use of TXA or Floseal^®^ was associated with less blood loss than that of the control group among patients undergoing primary TKA, as measured both directly (intraoperative bleeding + drainage) and on the basis of a decrease in Hb, without differences in the rate of complications. TXA and Floseal^®^ showed similar decreases in Hb and total measured blood loss, but the drain output was smaller in the Floseal^®^ group. Both interventional treatments exhibited comparable effects. The transfusion of blood components was infrequent in all 3 groups. Future investigations targeting the clinical importance of the differences found in the present study are necessary, as well as research investigating the effects of hemostatic and TXA in combination.

## AUTHOR CONTRIBUTIONS

Helito CP contributed to the intellectual concept of the study, performed surgeries, conducted the bibliographic research, evaluated and interpreted the data collected, and wrote the manuscript. Bonadio MB performed surgeries, evaluated and interpreted the data collected, and wrote the manuscript. Sobrado MF collected data, performed surgeries and drafted the manuscript. Giglio PN conducted the statistical analysis, drafted and revised the manuscript. Pécora JR analyzed the data collected, performed the final revision of the manuscript, and contributed to the intellectual concept of the study. Camanho GL analyzed the data collected, performed the final revision of the manuscript, and contributed to the intellectual concept of the study. Demange MK analyzed the data collected, performed the final revision of the manuscript, and contributed to the intellectual concept of the study.

## Figures and Tables

**Table 1:** Inclusion criteria.

- age above 21 years;
- male or female gender;
- indication for TKA for any cause;
- ability to read and understand the informed consent form;
- no previous history of open surgery on the knee (arthroscopy was accepted);
- absence of inflammatory arthritis (e.g., rheumatoid arthritis);
- absence of knee stiffness (defined as flexion less than 75 degrees or flexion contracture over 15 degrees) before surgery;
- absence of the following clinical conditions: kidney failure with creatinine clearance <40 mL/min, liver failure, severe heart failure, severe respiratory failure, history of thromboembolic events, coagulation disorders and previous stroke.

**Table 2:** Parameters for evaluation.

- Weight of 30 pads before and after surgery to assess the amount of blood absorbed during the intraoperative period
- Difference between the amounts of serum infused to irrigate the surgical field and the volume suctioned during the intraoperative period (suctioned blood volume)
- Output of the 3.2-mm vacuum drain (Portovac) until its removal (which occurred on postoperative day 2 as per protocol)
- Amount of fluids infused during surgery (colloid and crystalloid solutions)
- Decrease in the Hb level from the preoperative measurement to PO day 3
- Need for and number of blood transfusions, indicated when an Hb <8 g/dl was combined with the presence of clinical complications
- Occurrence of any thrombotic events with clinical manifestations within 90 days of surgery and clinical signs and symptoms of infection.
- Occurrence of any adverse events during the short-term follow-up that were likely to be related to the investigated medications
- Knee ROM during the first 3 days after surgery

**Table 3:** Mean values of the investigated variables related to blood loss.

	Aspirated blood volume (mL)	Difference in pad weight (g)	Number of transfused subjects	Drain output until PO 2 (mL)	Hb decrease on PO day 3 (g/dL)	Total measured bleeding (suctioned + pads + drain)
TXA	55.2*	197.5	1	546.5* ‡	3.03*	799*
Control	122.5* †	304.6	2	901.3* †	4.81* †	1328* †
Floseal^®^	59.9†	235.5	2	331.2† ‡	3.50†	626†

*,†,‡ *p*<0.05 in comparison with other values in the same column.

**Table 4:** Mean ROM of the knees assessed for the prospective randomized trial comparing Floseal^®^ and TXA for bleeding control after TKA.

Group	Control		TXA		Floseal^®^	
	Mean	SD	Mean	SD	Mean	SD
PO day 1	65.8*	20.1	73.5	19.2	80.5*	9.9
PO day 2	80.2* ‡	14.7	85.7† ‡	11.8	93.4* †	11.4
PO day 3	90.0* ‡	9.3	95.6† ‡	8.3	102.4* †	7.9
Difference between PO day 1 and 3	24.2	16.9	22.1	17.1	21.9	10.4

PO – postoperative.

*,†,‡ *p*<0.05 in comparison with other values on the same line.

TXA – Tranexamic acid.
